# Mobile Health Apps, Family Caregivers, and Care Planning: Scoping Review

**DOI:** 10.2196/46108

**Published:** 2024-05-23

**Authors:** Marjorie M Kelley, Tia Powell, Djibril Camara, Neha Shah, Jenna M Norton, Chelsea Deitelzweig, Nivedha Vaidy, Chun-Ju Hsiao, Jing Wang, Arlene S Bierman

**Affiliations:** 1 The Ohio State University College of Nursing Columbus, OH United States; 2 Montefiore Einstein Center for Bioethics Albert Einstein College of Medicine Bronx, NY United States; 3 Credence Management Solution USAID Global Health Technical Professionals Washington, DC United States; 4 National Institute of Diabetes and Digestive and Kidney Diseases Bethesda, MD United States; 5 Agency for Health Care Research and Quality Rockville, MD United States; 6 Center for Evidence and Practice Improvement Agency for Health Care Research and Quality Rockville, MD United States; 7 Florida State University College of Nursing Tallahassee, FL United States

**Keywords:** caregivers, carers, informal caregivers, family caregivers, mHealth applications, telemedicine, mobile health, mHealth, eHealth, digital health, apps, chronic condition, caregiver, application, support, clinicians, development, electronic health record, implementation, mobile phone

## Abstract

**Background:**

People living with multiple chronic conditions (MCCs) face substantial challenges in planning and coordinating increasingly complex care. Family caregivers provide important assistance for people with MCCs but lack sufficient support. Caregiver apps have the potential to help by enhancing care coordination and planning among the health care team, including patients, caregivers, and clinicians.

**Objective:**

We aim to conduct a scoping review to assess the evidence on the development and use of caregiver apps that support care planning and coordination, as well as to identify key factors (ie, needs, barriers, and facilitators) related to their use and desired caregiver app functionalities.

**Methods:**

Papers intersecting 2 major domains, mobile health (mHealth) apps and caregivers, that were in English and published from 2015 to 2021 were included in the initial search from 6 databases and gray literature and ancestry searches. As per JBI (Joanna Briggs Institute) Scoping Review guidelines and PRISMA-ScR (Preferred Reporting Items for Systematic Reviews and Meta-Analyses Extension for Scoping Reviews), 2 authors independently screened full texts with disagreements resolved by a third author. Working in pairs, the authors extracted data using a pilot-tested JBI extraction table and compared results for consensus.

**Results:**

We identified 34 papers representing 25 individual studies, including 18 (53%) pilot and feasibility studies, 13 (38%) qualitative studies, and 2 experimental or quasi-experimental studies. None of the identified studies assessed an intervention of a caregiver app for care planning and coordination for people with MCCs. We identified important caregiver needs in terms of information, support, and care coordination related to both caregiving and self-care. We compiled desired functionalities and features enabling apps to meet the care planning and care coordination needs of caregivers, in particular, the integration of caregiver roles into the electronic health record.

**Conclusions:**

Caregiver needs identified through this study can inform developers and researchers in the design and implementation of mHealth apps that integrate with the electronic health record to link caregivers, patients, and clinicians to support coordinated care for people with MCCs. In addition, this study highlights the need for more rigorous research on the use of mHealth apps to support caregivers in care planning and coordination.

## Introduction

### Background

In 2020, between 17.7 and 40 million Americans were family caregivers of adults aged 65 years or older [1], defined as unpaid relatives, partners, or friends who assist persons in daily activities due to disease, disability, or other conditions. The need for family caregivers is projected to increase by 2030 with the older adult population and complexity of care increasing [2]. Many care recipients have multiple chronic conditions (MCCs) defined as the presence of 2 or more chronic physical or mental health conditions [3]. Over a quarter of the US adult population (27.2%) struggles with MCCs, with the highest prevalence (76.9%) among adults with both Medicare and Medicaid [3]. People living with MCCs are high users of care, including outpatient, emergency, inpatient, postacute, home, and long-term care, as well as prescription drugs [4]. People with MCCs account for 64% of all clinician visits, 70% of all in-patient stays, 83% of all prescriptions, 71% of all health care spending, and 93% of Medicare spending [5].

Complex care routines are common among patients with MCCs and often difficult for people living with MCCs and their caregivers to maintain, leading to avoidable adverse events, poor health outcomes, increased health spending, duplication of services, and polypharmacy [6]. The many challenges associated with care complexity and care planning add to the physical, psychological, and financial burdens associated with caregiving [7]. In fact, 14.5% of American caregivers have reported that they experienced mental health decline for at least half the days in a month [2].

Poor caregiver health and unmet needs have been widely documented and include mental and physical health concerns [8], unmet need for information on medication and care management to support the care recipient [7], limited access to supportive services [7], issues with communication across the care continuum [9], and burdens associated with work, social isolation [7], and finances [10]. Importantly, assistance with care coordination and planning has been consistently noted as an unmet need for caregivers [11].

### Care Planning and Care Coordination

Developing care plans and organizing care involves the marshaling of personnel and other resources needed to carry out essential patient care activities and requires the exchange of information among participants responsible for different aspects of care [12]. Care planning is a collaborative process focused on discussing patient and clinical goals of care, conducting shared decision-making to identify strategies for clinical and self-management to achieve these goals based on evidence and patient preference, clarifying roles for different members of the care team, and empowering patients and caregivers [13]. These processes link health professionals, caregivers, and patients in the tasks of designing and implementing care.

Developing a comprehensive care plan both requires and supports care coordination by aggregating and streamlining data on health and social concerns, goals, care management strategies, and health status. Effective care coordination entails the organization of patient care activities to facilitate the appropriate and timely delivery of health care services by multiple clinicians in multiple care settings [12]. Care coordination involves the patient, clinicians, health care teams including nurses, pharmacists, physical therapists, and social workers, and caregivers. Such care coordination has been shown to benefit multiple domains, including decreased symptoms and mortality, and increased quality of life [14].

### Digital Solutions

Digital solutions offer an opportunity to alleviate some of the care planning and coordination burdens currently shouldered by caregivers and patients. Digital health solutions encompass a variety of information or communication technologies applied to health needs. Digital health is mobile health (mHealth) when implemented on mobile devices. Digital health apps—or programs designed to accomplish specific tasks—fall into the category of mHealth when they are designed to operate on a mobile device.

mHealth apps have the inherent capability of increasing the reach of interventions, and transcending geography and time. They are also often more broadly accessible in the United States, as the uptake of mobile devices is greater than desktop computers [11]. Furthermore, they can be explicitly tailored to individual needs. Recent advances in technology and software now allow apps to be linked to other digital devices and the electronic health record (EHR).

Several systematic reviews outlined challenges associated with existing apps for caregivers, especially insufficient scientific evidence to support the efficacy of these apps [15-20]. However, no review has focused either on care planning and coordination apps overall or on caregivers of people with MCCs. Moreover, no review focused on the importance of care planning and coordination between the caregiver, care recipient, and professional health care providers. We conducted a scoping review to examine the evidence on the development and use of caregiver apps designed to support care planning and coordination, identify key factors related to their use (ie, needs, barriers, and facilitators), and characterize desired functionality. This review was undertaken to inform the development of a comprehensive, interoperable electronic care plan with clinician-, patient-, and caregiver-facing components to enhance care planning and coordination, address fragmentation of health care, and enhance the collection and sharing of critical patient-centered data across community, clinical, and research settings for people living with MCCs. The Agency for Healthcare Research and Quality (AHRQ) and the National Institute of Diabetes and Digestive and Kidney Diseases (NIDDK), with support from the Assistant Secretary for Planning and Evaluation’s Patient-Centered Outcomes Research Trust Fund, are working in partnership to develop an interoperable e-care plan.

## Methods

**Figure 1:**
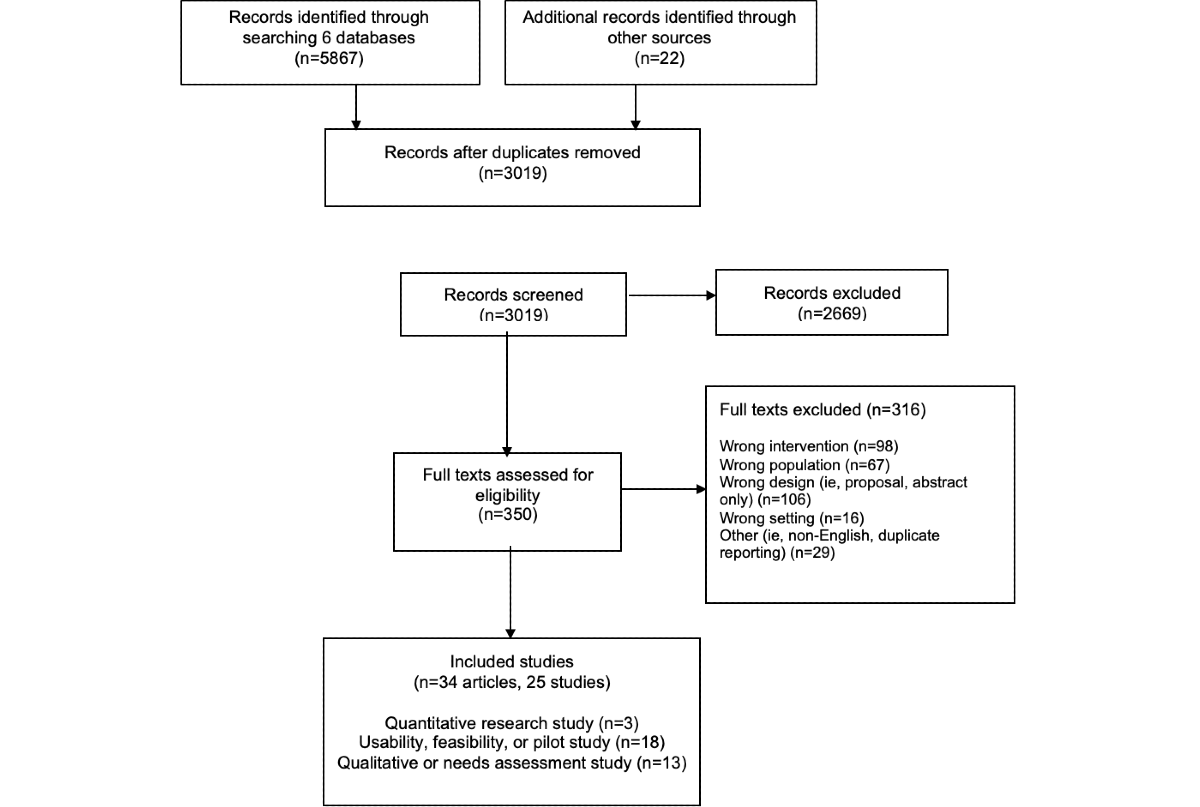
PRISMA flow diagram (adapted from Tricco et al [22], with permission from PRISMA). PRISMA: Preferred Reporting Items for Systematic Reviews and Meta-Analyses.

We conducted a scoping literature review using JBI (Joanna Briggs Institute) Scoping Review guidelines [21] and the PRISMA-ScR (Preferred Reporting Items for Systematic Reviews and Meta-Analyses Extension for Scoping Reviews) [22] to guide our methods and reporting. Papers published in English between January 2011 and June 2021 were included. Hence, our initial search activity specific to care planning and coordination revealed a dearth of papers, we broadened our search to include papers intersecting 2 major domains: mHealth apps and caregivers (Figure 1). We hoped to capture available information relevant to care planning and coordination from the perspective of the caregiver. We included mobile health apps like native apps (ie, residing on smartphones) as well as web-based apps designed for smartphone formats. We included all diseases and conditions and care settings (eg, ambulatory, hospital, home, hospice, and long-term care). Study types included pilot and feasibility and experimental and quasi-experimental study designs. Source documents included academic peer-reviewed journals, dissertations and theses, government policy documents, and white papers published by caregiver advocacy organizations (eg, AARP [American Association of Retired Persons] and National Alliance for Caregiving). Studies including paid caregivers or caregivers of patients aged younger than 18 years were excluded. Interventions delivered via social media, phone calls (including interactive voice response), video, telehealth, or text messaging alone were excluded. We also excluded interventions delivered in low- and middle-income countries given significant differences in information technology infrastructure and patterns of use [23]. As such, comparisons would be difficult. Research interventions involving assistive technologies (ie, motion sensors), non–health related, and health literacy alone were excluded. Source documents such as opinion or editorial papers, conference posters or abstracts, study protocols, blogs, and websites were excluded. Key search terms (Textbox 1) alone or in combination, were used to create our search protocols in 6 databases: PubMed, Cochrane, CINAHL, SCOPUS, Web of Science, and Embase. We conducted ancestry searches of caregiver app reviews and caregiver literature reviews and searched several domain-specific journal databases including the *Journal of the American Medical Informatics Association*, *Journal of Medical Internet Research*, *International Journal of Medical Informatics*, *Journal of the American Medical Association*, and *New England Journal of Medicine*.

**Search terms.**

**Caregiver**

- Caregiver; caretaker; care provider; carer; care

**mHealth app**

- mHealth; “mobile health” app; applications; “digital application”; eHealth; and smartphone
- Medical Subject Headings terms: telemedicine [encompasses mHealth]; mobile applications

We exported search results into EndNote, a reference management software platform to eliminate duplications, then uploaded them into Covidence, a web-based systematic review platform, to streamline evidence synthesis and author collaboration. Covidence allowed the research team to work collaboratively when screening papers at the title, abstract, and full-text level. In total, 2 authors independently screened titles and abstracts for eligibility with full-text screening conducted in the same manner. Screening disagreements were resolved through discussion or review by a third author. In keeping with scoping review methodological practices, critical appraisal, and risk of bias were not assessed.

Working in pairs, authors independently extracted data after adapting the JBI data extraction template and a previously used and pilot-tested data extraction table [24]. Then, each author compared results with the other for consensus about the extracted element. Data extraction elements included first author, publication date, health care domain of the care recipient, country, title, participant demographics, study purpose, study design, intervention description, app name and hyperlink if available, primary app users (ie, patient, caregiver, health care provider, and other), key or primary findings, app features and functionality—including desired functionality, how app supported care coordination, and how app supported caregivers (Multimedia Appendix 1 [25-45]). For qualitative studies, we extracted data elements associated with caregiver needs and desires. We used conventional content analysis methods, previously described by Hsieh and Shannon [46], to code and group categories as the phenomena of interest was new with little of the theoretical or literature available to guide the analysis. In keeping with conventional content analysis methods [47], we relied on inductive category development as categories and subcategories emerged from the literature, followed by deductive category and subcategory assignment.

## Results

### Overview

Of the 3019 nonduplicative records screened, 34 papers [25-45,48-60] representing 25 individual studies were included in this scoping literature review (Figure 1; Multimedia Appendix 1). Publication dates ranged from 2015 to 2021, with 29 (76%) papers published between January 2018 and August 2021. In total, 18 (53%) papers were feasibility, usability, or pilot studies [25-27,29-37,39-43,45] with qualitative or needs assessment papers representing 38% (n=13) [48-60]. Only 3 papers [28,38,44] reported using quantitative research methods to assess intervention efficacy (Textbox 2). Research was predominantly conducted in the United States (22 of 34). Further, 5 papers were from Australia, 3 from Spain, and one each from Canada, the United Kingdom, South Korea, and Turkey. In total, 14 papers focused on cancer caregiving, 7 on dementia caregiving, 6 on general caregiving, 2 each for stem cell transplant and mental health, and one each on heart failure, liver, mental health, and hospice. See Textbox 2 for details of the health care domain and paper type.

**Number of articles by health care domain and study type.**

**Cancer (n=14)**

- Experimental and quasi-experimental (n=1)
- Pilot, feasibility, or usability (n=9)
- Qualitative (n=4)

**Dementia (n=7)**

- Experimental and quasi-experimental (n=1)
- Pilot, feasibility, or usability (n=2)
- Qualitative (n=4)

**General caregiving (n=6)**

- Experimental and quasi-experimental (n=1)
- Pilot, feasibility, or usability (n=4)
- Qualitative (n=1)

**Mental health (n=2)**

- Qualitative (n=2)

**Stem cell transplant (n=2)**

- Pilot, feasibility, or usability (n=1)
- Qualitative (n=1)

**Heart failure (n=1)**

- Qualitative (n=1)

**Liver (n=1)**

- Pilot, feasibility, or usability (n=1)

**Hospice pain management (n=1)**

- Pilot, feasibility, or usability (n=1)

### Experimental and Quasi-Experimental Studies

Of the 3 quasi-experimental or experimental studies [28,38,44], Park and colleagues [38] developed an app for caregivers focused on knowledge of dementia, communication, and coping. Ferré-Grau and colleagues [28] conducted a randomized controlled trial of an app intervention designed to promote caregiver mental health. Finally, research conducted by Uysal et al [44], used an app for caregivers of patients with cancer focused on caregiver self-care and education. Overall, these studies, like many mHealth interventions for caregivers, addressed important caregiver needs including quality of life. However, none of these apps linked to information in the EHRs or leveraged data standards to support interoperability of data across the care team, nor did the apps provide enhanced communication among caregivers and the health care team. None of the studies investigated or measured care planning or coordination.

### Pilot and Feasibility Studies

In total, 18 pilot and feasibility papers [25-27,29-37,39-43,45], representing 12 studies, were included in this review. The majority (n=14, 78%) of these studies used small convenience samples. Furthermore, 11 of the papers focused exclusively on caregiver mental health or included a component of caregiver mental health in the interventions [25-27,32-34,36,37,39,40,43]. In total, 5 reported on apps that included disease education or caregiving education [25,26,31,37,41]. Further, 3 focused on caregiver communications with family and friends [41,42,45] but did not assess care coordination or communication with health care providers. One included education on the skills necessary to communicate with health care professionals but did not assess care planning, coordination, or communication as an outcome as it was a feasibility study [45].

Most of the pilot and feasibility studies focused on the important goal of supporting caregivers’ wellness but did not address care planning or coordination. For example, in one study—with results described in 3 papers [27,39,40]—the researchers conducted a 12-week feasibility study using a psycho-educational intervention delivered via video sessions with a goal of caregiver stress reduction. In another study [25,37], investigators used a mindfulness app and assessed cultural sensitivity and barriers to use as feasibility criteria. Kubo and colleagues [32-34] evaluated a commercially available mindfulness app to assess the feasibility of use to improve caregivers’ mental health. Similarly, Sikder and colleagues [43] pilot-tested an app focused on improving depression symptoms among caregivers.

In total, 7 papers included caregivers only as participants [27,31,36,39,40,42,43], while 9 papers included caregivers and care recipients as participants [25,29,30,32-35,37,41]. Only 2 feasibility studies, one conducted by Brown et al [26] and the other conducted by Wittenberg and colleagues [45], also included health professionals as participants. Brown and colleagues [26] examined the feasibility of an app for dementia caregivers, and included caregivers, homecare case managers, and primary health care providers as participants. The platform, CareHeros, was designed with the goal of bidirectional sharing of care recipients’ information between caregivers and health care professionals. The platform did not communicate with EHRs, and bidirectional communication was only reported between case managers and primary care providers, exclusive of caregivers and care recipients. There was limited uptake of the app, with participants logging into CareHeros an average of only 2.18 times over the 11-week period of this study. Wittenberg and colleagues [45] demonstrated the feasibility of an mHealth app to support caregiver communication skills related to caregiving. The overall objectives of the app development included: (1) to improve caregiver communication skills related to caregiving, (2) to facilitate information sharing among family members, (3) to provide self-care resources for caregivers, and (4) to increase caregiver knowledge. The app was not designed to connect to the EHR, nor was it designed to increase or support communication between caregivers and health care professionals. Caregivers and health care professionals participated in the design and the development of the app as well as usability and acceptability testing. Both groups found the app to be usable and acceptable for helping caregivers with educational needs and communication skills related to caregiving.

While none of the 18 pilot and feasibility studies directly evaluated care planning or coordination as an aim or outcome, 2 [30,35] investigated apps that could assist in care delivery—with caregivers assessing care recipients’ pain [35] and caregivers assessing care recipients’ hepatic encephalopathy [30]. Ganapathy and colleagues [30] used the PatientBuddy app, which sent alerts with critical values regarding hepatic encephalopathy to dyads of patients and caregivers as well as clinicians to support care management, obtaining a positive impact reducing 30-day readmissions in a small cohort. Mayahara et al [35] conducted a pilot study using e-Pain Reporter, which assisted caregivers in assessing and managing the pain of family members in home hospice. The e-Pain Reporter was designed to provide information on patient pain and pain management to nurses in real time. However, this pilot study did not assess the communication aspect of the app.

In summary, among these pilot and feasibility studies, heterogeneity in study design, interventions, and outcomes preclude meta-analysis, generalization, and direct comparisons. Additionally, most failed to provide support for care planning or coordination and none linked with the EHR or leveraged interoperable data standards. As with most pilot and feasibility studies, these results were preliminary, not powered to identify statistically significant differences in outcomes, and were specific to the app under investigation. Still, a small number of promising studies [26,30,35,45] attempted to enhance communication or information sharing, a component of care planning and coordination.

### Qualitative Studies

In total, 13 (38%) papers [48-60] included in this review were qualitative studies assessing caregiver needs associated with mHealth apps. These caregiver needs were synthesized into 3 broad categories: (1) needs associated with providing care, (2) needs associated with self-care, and (3) desired app features and functionality. In terms of providing care (category 1), caregivers needed information, support, and help with care coordination. For self-care (category 2), caregivers reported a need for information and support. A detailed list of desired mHealth app features and functionality (category 3) is provided in Textbox 3.

**Qualitative outcomes—caregiver needs.**

**Needs associated with providing care**

- **Information**
  Adjusting to a new role
  Information on disease or condition of care recipient
  Information on disease or condition common comorbidities
  Symptom, behavior, or safety
  When to seek help
  Changing nature of caregiving
  Financial and legal services (financial assistance, job help, and health care payment)
  On-demand education and training
  Community support links (transportation or community reintegration)
  Content tailored to care recipients’ needs
  Simple—easy to understand
  Up-to-date scientific evidence and mechanism for updating the information
  Multimodal delivery of information: video, audio, text, or animations
  Always accessible
- **Support**
  Support for care recipients’ physical and emotional needs
  Support with rehabilitation and activities of daily living (oral, bathing, dressing, grooming, toileting, feeding and nutrition, transferring, and ambulation)
  Decision-making support
  Medication management
  Tracking and monitoring of care recipient—mental, physical, emotional, and social (including symptoms, vital signs)
  Content tailored to care recipient’s needs
  Family or personal relationships (asking for help, safety, and communication)
  Always accessible
- **Care coordination**
  Integrated app with health care system—care coordination
  Ability to complete questionnaires at home, unrushed
  Finding care equipment
  List of important contacts and contact information for quick reference
  Information and connection to support services (specialty care, first responders, advocacy organizations, and respite services)
  Relationships with health care providers (personal contact)
  Feedback from health care providers—instant
  Automated data entry and reminders or prompts
  “One-stop-shopping”—all information in 1 place

**Needs associated with self-care**

- **Information**
  Information to help improve caregivers’ health (stress management, peer support, and support groups)
  Activities, programs, and therapy to improve mental, physical, and social support of caregivers
  Content tailored to caregivers’ needs
  Family or personal relationship help (safety or asking others for help or support)
  Preventing social isolation
- **Support**
  Tracking and monitoring of caregiver—mental, physical, emotional, or social (including symptoms, mental health, vital signs)
  Content tailored to caregiver needs
  Social media—“people like me” with expert moderator
  Peer mentor, support, or coaching

**Desired mHealth app features and functionality**

- Easy to use
- Easy to learn
- Integrated with phone contacts and other apps (exercise and weight management)
- Ability to report care recipient status or symptoms to health care providers and get a response, feedback, or follow-up quickly
- Task reminders (appointments, medication management, etc)
- Integrate with other platforms or devices (electronic health records, smart watches, or pharmacy)
- Share information with family members
- Integrate music or other entertainment
- Track patient symptoms or issues over time
- Track caregiver issues over time
- Customizable
- App from a trusted source and evidence-based content
- Data secure
- Integrated across health care systems
- Not too much information—just in time with the right information
- Affordable
- Font or screen size readable—Americans with Disabilities Act Standards for Accessible Design compliant
- Sustainable
- Help for digital naïve
- Does not reduce time with physician
- Clear perceived benefit
- Ability to personalize features and functions
- Automated data entry

## Discussion

### Principal Findings

This scoping review synthesized the evidence on the development and use of caregiver apps designed to enable or support caregiver participation in care planning or care coordination. We identified key factors (ie, needs, barriers, and facilitators) related to care planning and coordination. We described important functionalities and features enabling caregiver apps to meet care planning and coordination needs and facilitate caregiving activities. This comprehensive summary of caregiver needs related to health apps and care coordination may be useful to developers and researchers as it relates to caregivers of those living with MCCs. A better understanding of usability and overall needs will enhance ongoing research efforts to improve e-care planning and care coordination among these populations.

Of the 34 papers, representing 25 individual studies included in this review, only 3 were experimental or quasi-experimental intervention studies [28,38,44]. None of the studies included in this review focused on care planning, care coordination, or care recipients with MCCs. This paucity of research precluded generalizations about caregivers’ apps, much less in care planning and coordination. Although most of the studies included in this review addressed important caregiver factors including caregiver education, coping, and self-care, these standalone interventions lacked components to reduce caregiver burdens associated with planning and coordinating complex care. An app designed to specifically improve care planning and coordination, thus reducing this burden, is needed—particularly for the increasing number of care recipients with MCCs.

Most studies within this review were qualitative studies or pilot and feasibility studies. Yet, a few of these studies [26,30,35,45] identified elements important for care planning and coordination in mHealth apps. By definition, these studies are preliminary in nature thus precluding generalizations; they do not represent proven efficacy or settled science. However, they provide a foundation for future exploration of the role of mHealth interventions in promoting care planning and coordination.

### Comparison to Prior Work

Our findings parallel and extend the results identified in a recent review focused on native apps for informal caregiving [61]. Native apps are apps residing on smartphones as opposed to web-based apps. The principal findings specific to native apps [61] align with our more comprehensive review (including both native and web-based apps) in that the nascent technology has not matured enough to make meaningful recommendations beyond that of caregiver needs and wants. More rigorous research is needed, specifically among caregivers of patients with MCCs.

In terms of caregiver needs associated with care planning and coordination, caregivers and care recipients in included studies identified several important areas of needs and wants including apps that delivered “one-stop-shopping” or all the information in 1 place. These needs and wants were similar to those identified by Margarido and colleagues [61] in their 2022 scoping review. The results from both indicated caregivers wanted apps that integrated with the health care system (including the EHR) and could allow them to complete questionnaires at home in an unrushed fashion. They wanted apps that could help them find care equipment and information about support services and support contacts. Relationships with health care providers and feedback from the providers were of key importance, as were timely reminders and prompts (eg, upcoming appointments and medication changes).

### Future Directions

More research is needed as this scoping review did not identify any of the following: an app designed to provide access and enhance communication among caregivers, patients, and health care workers, with access for all 3 groups to the EHR; use of data standards in apps to promote interoperability of data across the care team, including caregivers and care recipients; a focus on care planning and coordination; a free and publicly available digital platform; or demonstration of successful usability, efficacy, and sustainability.

The potential exists for emerging mHealth apps to contribute to care coordination by linking caregivers, patients, and clinicians to information and resources that improve the ability of the entire care team to actively engage. Ongoing research focused on developing and evaluating [62-65] interventions to support caregiver engagement in health care through direct EHR access and other digital means could provide important insights. Today, mHealth app-facilitated care planning and coordination remains a possibility, not a reality. This scoping review provides further evidence that existing caregiver-facing mHealth apps are not sufficiently supported by research, with many studies focused on well-educated, tech-savvy female caregivers [30,37,50]. There is a need for app development to meet caregiver needs in diverse populations. Most such apps address the burdens of caregiving through interventions aimed at education, self-care, and stress reduction. Though these are helpful, they do not address the fundamental challenges related to care planning and coordination.

Current government federal policies encourage care planning and coordination. There is a federal mandate through the Office of the National Coordinator for Health Information Technology for third-party mHealth apps to integrate with the EHR. These technologies need to be implemented into current health care workflows, but data blocking and the inability to write back to the EHR present challenges. Current workforce shortages, especially for nurses, are well documented and may increase the difficulty of introducing new technologies and tasks, requiring both additional training and time from an already overburdened workforce. On the other hand, a well-designed app that facilitates information sharing, care planning, and communication could potentially reduce the burden.

### Strengths and Limitations

We acknowledge several limitations of this study. First, papers included in the scoping review do not include work published after June 2021. It is possible our search terms failed to identify relevant papers in this rapidly developing field. Second, the review included only studies published in English. Though digital health literature is predominantly published in English, there is the possibility of missing important work in other languages. In keeping with guidelines for scoping reviews, we neither assessed the risk of bias nor methodologies in the included studies. Finally, the heterogeneity of included research precluded a meta-analysis of findings across all studies.

### Summary

This scoping review synthesizes the current evidence on developing mHealth apps to support caregivers in care planning and coordination, providing insights to inform future mHealth app development to engage caregivers as members of the health care team, share critical information across the entire health care team, reduce the burdens caregivers experience in trying to coordinate care, as well as identifying the functionality caregivers desired. Few experimental studies involving apps with needed functionality were identified in the scoping review, even though use of digital technology for caregiver support is a growing interest. We found no studies focused on care planning or coordination, and a very small number of pilot and other preliminary studies addressing specific aspects of care coordination, such as communication. Given the limited number of studies and the preliminary nature of many, there is insufficient evidence on mHealth apps to support caregivers in care planning and coordination. However, the need and potential for further work to achieve these aims is substantial.

### Conclusions

In sum, research and evidence on the effective use of mHealth apps to support caregivers involved in care planning and coordination for people living with MCCs is limited. Apps to support caregivers have yet to be integrated into the EHRs. Multidirectional communication between caregivers, care recipients, and health care providers through the EHR holds great promise for relieving the burden on clinicians, patients, and their caregivers alike. The development and implementation of an mHealth app linking the 3 key stakeholder groups to work together to [65] enhance care planning and coordination, remains an unmet need. Prior work on the functionality desired by caregivers can inform this work.

## Appendix

Multimedia Appendix 1 Quantitative and pilot, feasibility, or acceptability study information.

Multimedia Appendix 2 PRISMA-ScR checklist.

## Abbreviations

AARP: American Association of Retired Persons
AHRQ: Agency for Healthcare Research and Quality
EHR: electronic health record
JBI: Joanna Briggs Institute
MCCs: multiple chronic conditions
mHealth: mobile health
NIDDK: National Institute of Diabetes and Digestive and Kidney Diseases
PRISMA-ScR: Preferred Reporting Items for Systematic Reviews and Meta-Analyses Extension for Scoping Reviews
