# Supplementary material for: Mobile Health Apps, Family Caregivers, and Care Planning: Scoping Review
Source: J Med Internet Res. 2024 May 23;26:e46108. doi: 10.2196/46108 (PMC11157180; doi:10.2196/46108)
Supplement: Multimedia Appendix 1 [file jmir_v26i1e46108_app1.docx]

**Multimedia Appendix 1.** Quantitative and pilot, feasibility, or acceptability study information.

| **Author, Date, Country** | **Study Purpose** | **Study Design or Focus** | **Participant Number (N=#), Mean Age (years), Female %** | **App Name** | **Potential app users:**  **P-patient or care recipient, C-caregiver, H-healthcare provider,**  **O-other** | **Key findings** | **Support for care team or care coordination** | **Support for caregiver** | **Electronic health record integration** |
| --- | --- | --- | --- | --- | --- | --- | --- | --- | --- |
| **Quantitative Research Studies** | | | | | | | | | |
| Ferre-Grau, 2021, Spain | To evaluate the effectiveness of an app-based intervention to improve caregiver mental health and assess app satisfaction. | Two-arm RCT | App (N=56), control (N=57), 92% | TIVA | C | At 1 month: no statistical difference between groups on any measure. At 3 months, intervention group had statistically significant improvement in mental health and caregiver burden. App satisfaction was high. | NA | Decreases depressive symptomatology | No |
| Park, 2020, South Korea | To evaluate the effect of a 4-week app intervention compared to a handbook intervention in managing behavior and psychological symptoms of home-dwelling patients with dementia. | Nonequivalent, two-group, pre- post-test | App ( N=12), 54.5, 66.7%; Handbook intervention group (N= 12), 61, 50% | CMAP | C | Statistically significant decrease in fatigue in app group compared to handbook group. | NA | App provides education and emotional and support resources | No |
| Uysal, 2021, Turkey | To evaluate the effectiveness of a mobile-assisted empowerment program developed for caregivers of cancer patients undergoing radiotherapy. | Two-arm RCT, with pre-, post-test | App (N=36), 49.6, 59.3%; standard care control (N=38), 46.6, 55.3% | NA | C | Compared to baseline, app users’ mean distress scores were significantly lower than the control groups. Quality of life was also higher in the intervention group. | NA | App provided 3 educational modules for caregivers: 1) radiotherapy symptom management, 2) caregiver care, 3) communication | No |
| **Pilot, Feasibility, and Acceptability Studies** | | | | | | | | | |
| Brown, 2016, U.S. | Evaluate the use and utility of an app for caregivers of Alzheimer’s patients | App Use and Utility | Caregivers (N=11), 56.5, 82%;  Case managers (N=6), 36.4, 83%;  Primary Care Providers (N=5), 20% | CareHeroes | C, H, O (case managers) | All users reported satisfaction with the app. Caregivers: 70% agreed or somewhat agreed that navigation and tasks were easy to perform. | Theoretical potential to improve communication among team members as most content was viewable by caregiver, case manager, and primary care providers | Provides resources for caregivers related to services, information, emotional, and decisional support. | No |
| Beer, 2020, U.S. | Explore attitudes and acceptance of mindfulness app for lung cancer survivors and their caregivers | App Acceptability, Feasibility, and Usability | Caregivers (N=8), 58.6, 38% Cancer survivors (N=11), 64.6, 73% | Breathe Easier | C, P | Participants perceived over twice the benefits than concerns. Survivors identified more benefits than their caregivers. | NA | Mindfulness and disease education | No |
| Owens, 2020, U.S. | Assess cultural sensitivity of app | App Acceptability | Caregivers (N=6), Cancer survivors (N=6), all African American, total mean age = 59 | Breathe Easier | C, P | Assess cultural sensitivity of app | NA | Mindfulness and disease education | No |
| Carr, 2019, U.S. | Describe caregivers’ perceptions of usability and acceptability and identify future adaptations | App Usability and Acceptability | Caregivers intervention interaction (N=26), 53.3, 73%; Caregiver perceptions (N=14), 52.5, 71% | Pep-Pal | C | Disease information was most helpful, session lengths of 10-20 min. were appropriate. Mobile app format over web format was easiest, app was easy to use and helped improve self-care, guild, isolation, latent traumatizing effects. | NA | Designed to reduce caregiver stress | No |
| Pensak, 2017, U.S. | Formative evaluation to adapt and enhance app focused on caregiver stress management | App (anticipated) Usability, Feasibility, and Acceptability | Caregiver focus group (N=6), 44-66, 100%; Formative evaluation:  Healthcare Professionals (N=20), Caregivers & Patients (N=9); Caregiver interviews (N=6) | Pep-Pal | C | Content was linked to usability, acceptability, and feasibility; areas for app improvements were identified, and modifications were made to the app. | NA | Designed to reduce caregiver stress | No |
| Pensak, 2021, U.S. | Pilot study to examine usage rates and preliminary efficacy of anxiety, depression, stress, and sexual dysfunction | Pilot, 2-arm | Caregiver app users (N=26), 53.3, 73%; Usual care control (N=30), 55, 76% | Pep-Pal | C | App group experienced greater reductions in perceived stress, ability to manage stress, and for women, increased sexual functioning when compared to the control group; no difference in anxiety or depression; minimum usage rates criteria for success were not met. | NA | Designed to reduce caregiver stress | No |
| Frisbee, 2016, U.S. | Identify factors and characteristics of veterans and their caregivers that predict use of mHealth apps | Pilot, descriptive study | Veteran/family caregiver dyads (N=882),  Caregivers, 40, 94.9%  Veterans, 39, 4.3% | Launchpad App | C, P | Computer skills, living in a rural location, low levels of caregiver preparedness, veterans with mental health diagnoses, veterans’ age, and being a caregiving spouse were associated with app use. | Theoretical, not evaluated | Combines a suite of apps focused on caregivers and veterans’ care. | No |
| Ganapathy, 2017, U.S. | Evaluate the feasibility of using a mHealth app focused on cirrhosis and gauge the impact on 30-day readmission rates. | Pilot study, proof-of-concept, and feasibility | Caregivers (N=40)  Patients, (N=40), 58, 40% | Patient Buddy | C, P, H | Most patients and caregivers reacted positively to the app's educational content. Technical barriers and interference with daily life were negatives. 8 patients used the alert function after office hours for app instructions or to note changes. 8 potential readmissions were prevented. 15% did not use app as it was too demanding and required too much information. | Theoretical potential to avert readmissions | No direct caregiver support – caregivers enter cognitive and physical health assessments of the care recipient. | No |
| Heynsbergh, 2019, Australia | Design a prototype app for caregivers of adults with cancer | Focus Group and Interviews with caregivers, app, acceptance, and user experience testing | Interviews (N=33), focus group (N=12), 55, 60%  App testing-convenience sample (N=10), 3 younger than 30, 3 between 30-49, 5 were 50 or older, 50% | Carer Guide App | C | Attitudes of carers – app might improve ability to seek information and support their health and the care recipient’s health. A variety of information to be included in the app was identified, including information and caregiver support. App was user-friendly, but users had technical issues. Project time constraints and inability to meet all caregivers’ needs were limits. | NA | Caregiver wellbeing information | No |
| Kubo, 2018, U.S. | Assess the feasibility of using a commercial app to reduce caregiver and cancer patient stress | Feasibility (adherence, retention) | Caregivers (N=9), 58.8, 77.8%  Patients (N=19), 64.7, 68.4% | Headspace | C, P | 116 patients contacted, 28 enrolled, 19 completed (9 caregivers completed, 14 enrolled). Of those completing the study, 71% practiced meditation >50% of the days, with 64% meditating once a month after the intervention ended. | NA | Mindfulness practice | No |
| Kubo, 2019, U.S. | Assess the feasibility of conducting an RCT and examine the preliminary efficacy of a commercially available mindfulness-based app to reduce stress and improve quality of life (QoL) | Feasibility and preliminary efficacy, RCT | Caregiver Intervention (N=17), 57.1, 52.9%, Caregiver Control (N=14), 58.2, 64.3%  Patient Intervention (N=54), 59.3, 62.3%, Patient Control (N=43), 56.7, 76.7% | Headspace | C, P | 74% of patients and 84% of caregivers completed the study. Among intervention participants, 65% practiced at least 50% of the days during the intervention period.  QoL improved among intervention patients compared to controls. No change among caregivers. Feasibility of conducting an RCT was demonstrated. | NA | Mindfulness practice | No |
| Kubo, 2020, U.S. | Assess the feasibility of conducting a cluster RCT comparing mindfulness-based interventions (app, web-based, waitlist control group) | Feasibility of conducting a cluster RCT | Caregivers (N=39), 66, 79%  Patients (N=103), 67, 70% | Headspace | C, P | Nearly all chose the app. 68% of patients and 47% of caregivers practiced mindfulness at least 50% of the days of the intervention.74% of participants were very or extremely satisfied with the program. Anxiety, QoL, and mindfulness improved among patients when compared to controls. | NA | Mindfulness practice | No |
| Mayahara, 2019, U.S. | Assess the feasibility of caregiver use of a pain reporting app, describe patient pain characteristics, and caregiver self-efficacy and barriers to app use | Feasibility of use and descriptive study | 12 caregiver-patient dyads.  Caregiver (N=12), 53.7, 91.7%  Patient (N=12), 67.8, 58.3% | e-Pain Reporter | C, P | Caregivers’ reports on patient pain were reviewed weekly. Over 9 days of use, caregivers reported pain on average 5.89 days. Satisfaction score was 2 on a 0 to 3-point Likert scale. Participants found the app easy to use. | NA | Help caregivers manage hospice patient’s pain | No |
| Otero, 2020, Spain | Evaluate feasibility and preliminary efficacy of a depression prevention app for caregivers | Single arm, pre-post-test | Caregivers (N=31), 54, 93.5% | Happy | C | 93.5% intervention adherence and all attending weekly conference call sessions. App use not reported. Participants reported overall satisfaction with the intervention. Incidence of depression, depressive symptoms, and risk of depression were decreased. | NA | May decrease risk of depression and depressive symptoms | No |
| Quinn, 2019, U.S. | Determine the usability of a commercially available app and network platform by older adults and their caregivers | Observational usability study | 8 caregiver-patient dyads, Caregivers, 54.8, 92%  Patients, 77.8, 67% | ICMed | C, P | Usability and engagement among participants were average. Older adults considered app features well integrated, while caregivers did not. Caregivers felt they needed to learn more before using the app, whereas older adults did not | NA | App supports information sharing and communication with family members | No |
| Quintana, 2019, U.S. | Assess the usability of a mHealth app/platform to manage medications | Think-aloud usability testing, surveys | Caregivers (N=7), 50, 71% | InfoSAGE | C | Thematic analysis revealed confusion among caregivers when entering dose and strength into the app. Entering medication information felt “redundant.” Confusion over side effects and drug-drug interactions occurred. Drug naming conventions (generic versus brand) proved challenging for participants. Other challenges were reported | Potential to enhance communication and medication management was not realized | Share medication management between patient and family members | No |
| Sikder, 2019, U.S. | Investigate the feasibility and limited efficacy of an app delivering mentalizing imagery therapy | Feasibility testing and changes in depression symptoms, mood, and caregiving experience | Caregivers (N=17), 66.52, 71% | NA | C | Usage – Usage patterns varied: low (N=6), moderate (N=6) & high (N=5). The app demonstrated technical feasibility, usage, mode improvement, and relationship understanding. Most participants found guided imagery and mindfulness exercises helpful. Depression scores improved with a large effect size for participants in the moderate and high-use categories. Mood improvement was also noted. Other caregiver-perceived benefits were noted. | NA | Mentalizing Imagery Therapy to improve caregiver mental health | No |
| Wittenberg, 2019, U.S. | To illustrate a mHealth app designed to support communication for informal cancer caregivers. | Acceptability testing of app prototype | Usability – Caregivers (N=5); Acceptability – Caregivers (N=6); Quality and impact on healthcare providers (N=26) | NA | C | Caregivers – easy to use and navigate, positive feedback on acceptability. Caregivers were more likely to use app if recommended by provider.  Healthcare providers – helpful in increasing caregivers’ knowledge, and awareness of caregiver needs, improving communication and encouraging caregivers to seek help and motivation to address caregiver concerns. Good functionality, performance, esthetics, and design. Lower ratings for repeat use, customization, and interactivity. | NA | Improved knowledge of communication | No |

*Like colors denote multiple articles related to a single app – white denotes individual article of one app.
